# Effects of traditional Chinese medicine combined with chemotherapy for extensive-stage small-cell lung cancer patients on improving oncologic survival: study protocol of a multicenter, randomized, single-blind, placebo-controlled trial

**DOI:** 10.1186/s13063-021-05407-1

**Published:** 2021-07-08

**Authors:** Yuyi Chen, Mingwei Yu, Zishen Liu, Yi Zhang, Qiwei Li, Guowang Yang

**Affiliations:** Department of Oncology, Beijing Hospital of Traditional Chinese Medicine, Capital Medical University, No. 23, Back Road of Art Gallery, Dongcheng District, Beijing, 100010 China

**Keywords:** Extensive-stage small-cell lung cancer (ES-SCLC), Traditional Chinese medicine (TCM), Randomized controlled trial (RCT), Overall survival (OS), Quality of life (QoL)

## Abstract

**Background:**

Extensive-stage small-cell lung cancer (ES-SCLC) is characterized by extensive metastases, aggressive progression, and poor prognosis. Chemotherapy is applied as a preferred first-line regimen for ES-SCLC, but inadequate for improving its overall survival. Traditional Chinese medicine (TCM) is widely used in the clinical practice of ES-SCLC for its synergy with chemotherapy. However, there is still no substantial evidence to prove that TCM can effectively improve the long-term efficacy of ES-SCLC patients. The study intends to determine whether the TCM with chemotherapy can improve the overall survival (OS) in treating with ES-SCLC when compared with chemotherapy alone.

**Method/design:**

A multicenter, randomized, single-blind, placebo-controlled clinical trial will be conducted to determine whether the TCM granules combined with chemotherapy can improve the OS of ES-SCLC. Two hundred seventy participants will randomly receive 4–6 cycles (21 days per cycle) of chemotherapy plus TCM granules or placebo. The primary outcome measure is OS. The secondary outcome measures includes progression-free survival (PFS), objective response rate (ORR), quality of life (QoL), and tumor markers. Visits will be performed at the end of each cycle during the treatment period and then every 3 months in the follow-up period until the patients’ death or study completion.

**Discussion:**

The study’s result will provide a high-level evidence for TCM granules using with chemotherapy on the first-line treatment of ES-SCLC.

**Trial registration:**

Chinese Clinical Trial Registry ChiCTR1900022991. Registered on 6 May 2019 (prospective registration).

**Supplementary Information:**

The online version contains supplementary material available at 10.1186/s13063-021-05407-1.

## Background

Small-cell lung cancer (SCLC) is an aggressive and rebellious neuroendocrine carcinoma, which accounts for approximately 12–15% of lung cancer [1]. More than half of the newly diagnosed SCLC patients have developed into the extensive stage (ES), with lesions beyond the boundaries of hemithorax, whose 2-year mortality exceeds 95% [2]. In the past three decades, platinum-based chemotherapy, the first-line regimen of ES-SCLC has stagnated in the median progress-free survival (PFS) of 9–11 months, which is far from meeting the cure needs [3]. Even if the chemo-periods prolonged or dosages enhanced, the limited improvement in PFS and the same overall survival (OS) would come at the expense of more severe adverse events [4, 5], probably leading to the interruption of treatment [6]. The landmark breakthrough appeared in the treatment of ES-SCLC until the IMpower 133 and CASPIAN, adding the immune-checkpoint inhibitors (ICIs) to the standard etoposide and platinum [7]. Although this combination also tends to a heavier treatment burden because of its overprices and immune-related adverse reactions [8, 9], it does inspire a strategy for combining multiple treatments to benefit more ES-SCLC, especially on the grounds of chemotherapy, as some studies have tried [10, 11]. Thus, it is of great value to enrich the first-line regimens of ES-SCLC if therapy with better practicability and cost-effectiveness can be found in cooperation with chemotherapy.

Through a series of meta-analysis, traditional Chinese medicine (TCM) has been shown to improve the efficacy and safety with chemotherapy in varieties of malignancies, such as breast cancer [12], colorectal cancer [13], and non-small cell lung cancer [14]. TCM regimens have also increasingly widely used in the clinical practice of ES-SCLC for its synergy with chemotherapy. In previous researches [15], the long-term and regular TCM regimens were preliminarily considered as a favorable prognosis factor of ES-SCLC. Unfortunately, due to the small sample size and substandard design, no study so far has provided a clear-cut answer to whether TCM regimen can synergize chemotherapy to achieve longer oncologic survival of ES-SCLC. Therefore, we organized a randomized controlled trial (RCT) with relatively high-quality to verify the prognostic value of TCM on ES-SCLC.

The primary objective of this RCT is to determine whether the TCM granules with chemotherapy can improve the OS in treating with ES-SCLC when compared with chemotherapy alone. The trial will also provide clinical evidence on the efficacy of TCM granules in progression-free survival (PFS), objective response rate (ORR), quality of life (QoL), and tumor markers of ES-SCLC, alongside a safety analysis.

## Methods/design

### Study design

**Fig. 1:**
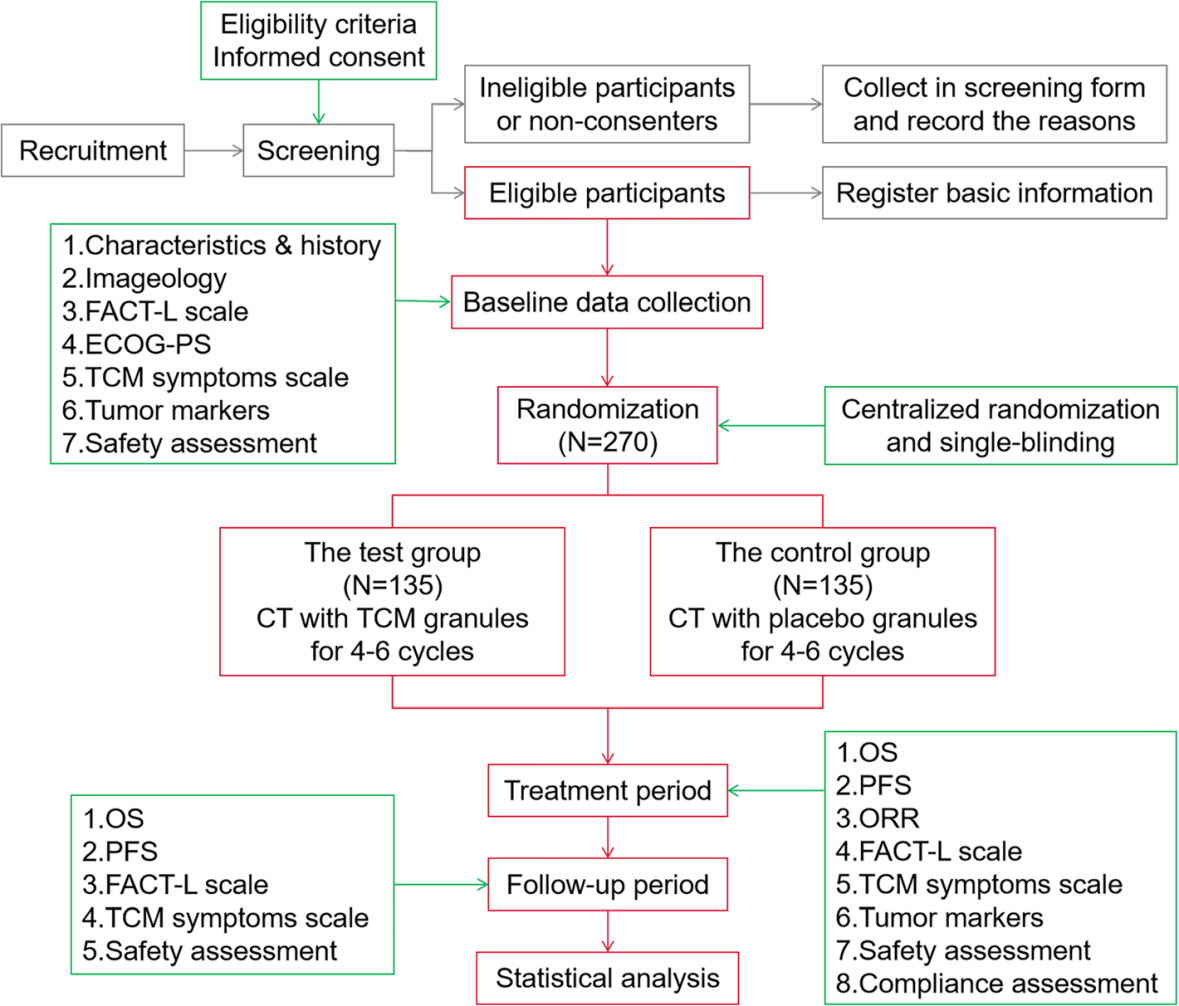
Flow diagram of study design. OS, overall survival; PFS, progression-free survival; ORR, objective response rate; ECOG-PS, Eastern Cooperative Oncology Group performance status; FACT-L, Functional Assessment of Cancer Therapy-lung carcinoma; CT, chemotherapy; TCM, traditional Chinese medicine

This is a multicenter, randomized, single-blind, placebo-controlled clinical trial, with superiority in the framework. After a run-in period within 7 days, 270 eligible participants will be randomized to the test group (TCM granules with chemotherapy) or the control group (placebo with chemotherapy) in a 1:1 ratio. All participants will undergo a treatment period of 12–18 weeks and a follow-up period until death. During the treatment period, both groups have to receive chemotherapy for 4–6 cycles. In the interphase of chemotherapy (days 6–21 of each cycle), participants in the test group will take TCM granules while those in the control group will take placebo granules for 15 days. Visits will be performed at days 0, 21, 42, 63, 84, 105, and 126 in the treatment period, and the last week of every 3 months (approximately 90 days) during the subsequent follow-up period. The study is scheduled to open from December 2018 to December 2021. The recruiting was started in June 2019 and lasted for 24 months. The endpoint of follow-up is the date of death or December 31, 2021 (Fig. 1).

### Settings and participates

Participants will be recruited from six general hospitals located in three different provinces in China, including Beijing Hospital of Traditional Chinese Medicine, Capital Medical University, Dongfang Hospital affiliated to Beijing University of Chinese Medicine, China-Japan Friendship Hospital, Longhua Hospital affiliated to Shanghai University of Traditional Chinese Medicine, Hubei Provincial Hospital of Traditional Chinese Medicine, and Beijing Hospital. Recruitment is done by posting advertisements or disseminating notices at the website and official WeChat of the six hospitals.

Inclusion criteria are as follows: (1) participants diagnosed with SCLC confirmed cytologically or histologically and have measurable lesions; (2) diagnosis of extensive-stage following the Veterans Administration Lung Study Group staging criteria; (3) participants who have not received surgery, radiotherapy, chemotherapy, or immunotherapy before; (4) an Eastern Cooperative Oncology Group performance status (ECOG PS) score of 0–2 points with expected survival over 3 months; (5) aged 18–75 years old; and (6) agree to sign of the informed consent voluntarily.

Exclusion criteria are as follows: (1) participants with dyspnea due to massive pleural, ascites, or pericardial effusion; (2) participants have symptomatic bone metastases and need to radiotherapy; (3) brain metastasis; (4) presence of pregnancy, lactation, and severe primary diseased in the heart, liver, kidney, or hematological system; (5) allergy to drugs used in this study; and (6) participants undergoing other clinical trials.

Withdrawal criteria are as follows: (1) antineoplastic proprietary Chinese medicine, TCM decoction, or injection outside of the protocol was used during the treatment period; (2) participants intend to receive radiotherapy, immunotherapy, and other antineoplastic therapies during the treatment period; (3) participants are no longer suitable to remain in the trial because of their complications; (4) severe adverse events; (5) severe adverse events; (6) participants ask to withdraw from the trial for any reason; (7) ineligible participants who have registered by mistake must withdraw from the trial in time; (8) ineligible participants who have registered by mistake must withdraw from the trial in time; and (9) participants lost to follow-up. If participants meet the withdrawal criteria, the investigators should record the reasons on the case report form (CRF) and follow up periodically as possible until the endpoint.

### Intervention

The protocol interventions consist of chemotherapy and TCM regimens.

#### Chemotherapy

All the eligible participants will undergo 4–6 cycles (21days per cycle) chemotherapy with the standard regimens recommended by the National Comprehensive Cancer Network (NCCN) clinical practice guidelines in SCLC (Version 2, 2018), and the preferred one is EP (Etoposide 100 mg/m^2^ on days 1–3, Cisplatin 75 mg/m^2^ on day 1, every 21 days).

#### TCM regimens

The TCM regimens including six kinds of granules corresponding to the six most common types of TCM syndromes in ES-SCLC patients. As shown in Table 1, participants with indications of a syndrome type, including all pulmonary symptoms, tongue, and pulse, and more than two systemic symptoms, will be diagnosed with the type by TCM physicians and received the matched granules. Since participants’ symptoms are likely to change over time along with the treatment, the diagnosis of types will be redone before each cycle to determine which kind of granules is the most tailored one.

**Table 1:** Indications of TCM granules and placebo

Name of granules	Syndrome types	Indications		
		Pulmonary symptoms	Systemic symptoms	Tongue and pulse
TCM Recipe 1 Placebo Recipe 1	Qi-Ying deficiency syndrome	Cough weakly with little phlegm.	Fatigue, sweating, hot flashes, palpitations.	Reddish tongue with thin or less coating, thready pulse.
TCM Recipe 2 Placebo Recipe 2	Lung-spleen Qi deficiency syndrome	Cough and asthma with white dilute sputum.	oppression in chest, fatigue, loss of appetite, abdominal distension, dropsy, loose stools.	Plump tongue with tooth prints and white greasy coating, sunken and thready pulse.
TCM Recipe 3 Placebo Recipe 3	Lung-Yin deficiency syndrome	Dry cough without phlegm.	Hot flashes, sweating, thirsty, irritability, hoarse voice.	Red tongue with less coating, quick and thready pulse.
TCM Recipe 4 Placebo Recipe 4	Qi stagnation and blood stasis syndrome	Cough and dyspnea with phlegm difficult to cough up.	Stabbing or distending pain, subcutaneous hemorrhage, depression and anxiety.	Cyanotic tongue with thin coating, wiry and astringent pulse.
TCM Recipe 5 Placebo Recipe 5	Heat-phlegm obstructing lung syndrom	Cough with excessive yellow sticky phlegm.	Fever, oppression and pain in chest, hemoptysis, thirst.	Red tough with yellow greasy coating, quick and slippery pulse.
TCM Recipe 6 Placebo Recipe 6	Qi deficiency with excessive cancerous toxin syndrome	Cough is aggravating, and lesions in lung progressed.	Weight loss, fatigue, loss of appetite, chest pain, cancerous fever, hemoptysis, sweating.	Dark tough without luster, thick greasy or denuded coating, sunken and wiry pulse.

*Abbreviations: TCM* traditional Chinese medicine. The diagnosis criteria of a syndrome type is that the participant has its pulmonary symptoms, supporting evidence of tongue and pulse, and more than two systemic symptoms. The corresponding granules will be used in accordance with syndrome types and randomization

Participants in the test group will take TCM granules with 200ml warm water twice daily for 15 days from the sixth day of each chemotherapy cycle. The herbs and dosages of the TCM granules are shown in Supplement 1. While those in the control group will take placebo granules composed of 95% dextrin and 5% TCM granules of each type in the same way. Both the TCM granules and placebo are made uniformly in Beijing Tcmages Pharmaceutical Co. LTD (Shunyi District, Beijing, Datong Road, No. 103, 10098) to ensure the similarity in appearance, smell, texture, and taste. And the participants are required to return the drug boxes and labels to CRC as evidence of timely medication.

### Randomization and allocation concealment

Centralized randomization was carried out by Peking University clinical research institute (PUCRI) with using the REDCap system (Version 8.4.3), a remote electronic data capture (EDC). When an eligible participant is enrolled, the clinical research coordinator (CRC) has to register the detailed information of the participant on the REDCap website. Then, after the confirmation of the principal investigator (PI) or sub-investigator (Sub-I), the participant will be assigned to either the test or control groups in an allocation ratio of 1:1. The REDCap system will automatically generate the participant’s ID numbers and feedback the grouping results to PI or Sub-I, who will next inform the drug administrator to prepare TCM granules or placebo granules for the participant.

**Fig. 2:**
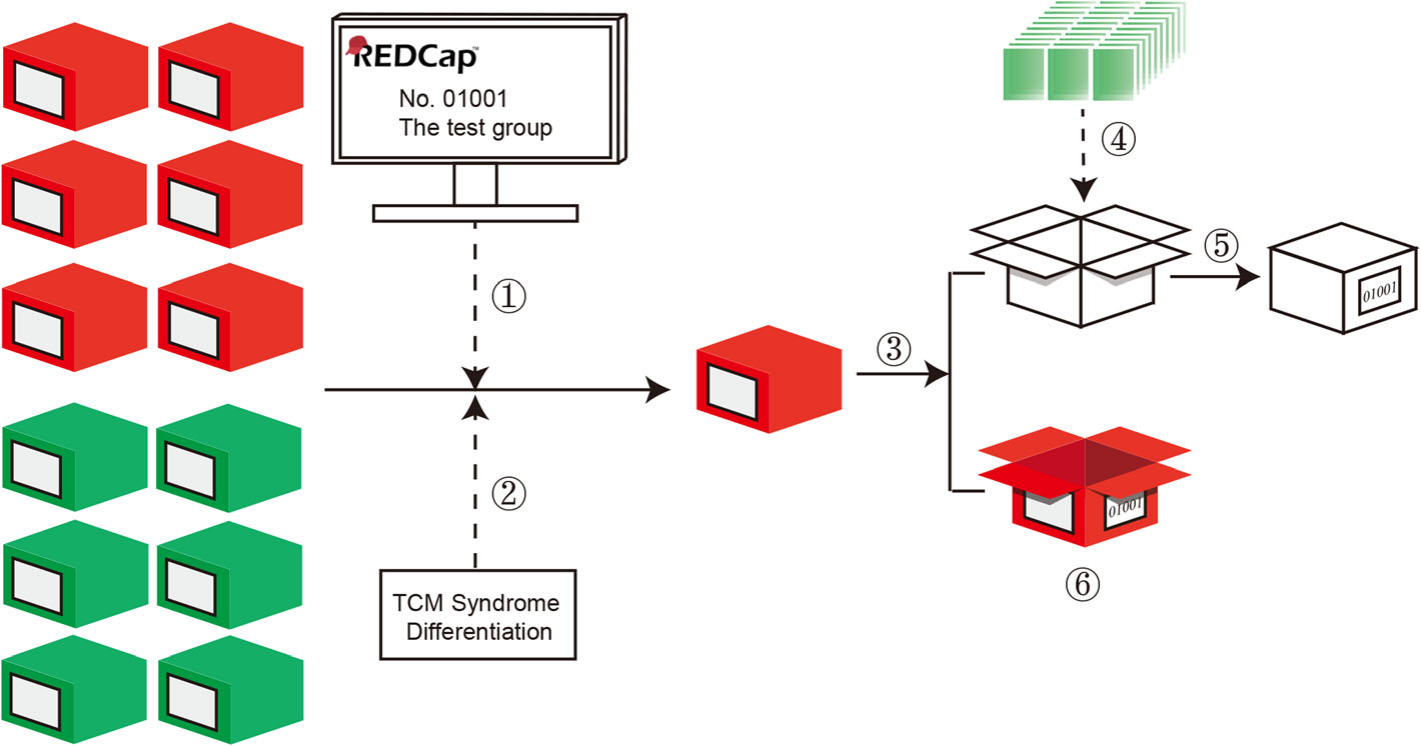
Procedure of single-blind. Take participant No.01001 as an example. (1) The participant was randomly assigned to the test group by REDCap system. (2) The participant’s TCM syndrome type was diagnosed by a TCM physician according to Table 2. (3) The drug administrator selects the correct TCM granules of corresponding type. (4) The sachets of TCM granules were removed from the red box to a white box. (5) The white box was labeled by No.01001 and passed to the participant, who was blinded until the end of the study. (6) The red box was labeled too and kept as evidence of single-blind by the drug administrator

Single-blind is used in the study (Fig. 2). After being informed of grouping and syndrome type, the drug administrator has to select the correct drug from storage, where the TCM granules are packaged in red boxes while the placebos in green. Then, he will remove the granules’ sachets from the red/green box to a white box and label the original red/green box and the current white box respectively with the same code that hides the information of the participant’s group and type. While keeping the red/green box as evidence of single-blind, the drug administrator needs to give the labeled white box to the investigator and finally pass them to the participant. The participants and statistical analysts are blinded until the trial is completed.

### Sample size calculation

The sample size was calculated by PASS 15.0 software. The model was established according to the log-rank test of survival analysis (bilateral side), with α=0.05 and power 1 – β = 0.8. Based on recent researches [15, 16], the median survival in ES-SCLC with first-line chemotherapy was approximately 8 months, which we estimated to be 4 months longer after TCM combined with chemotherapy. The recruitment period was set as 24 months while the total time was 36 months. The minimum sample size was calculated as 112 in each group. Considering a 10% loss, we sought to enroll at least 270 (135 per group) participants in the study.

### Assessments and time-points

Assessments will be performed at baseline and every protocol-defined interval (Table 2) until the occurrence of death or study completion.

**Table 2:** Schedule of data collection

	Screening and baseline	Treatment period						Follow-up period			
**Visits**	**--**	**1**	**2**	**3**	**4**	**5**	**6**	**7**	**8**	**9**	**……**
**Time points (days)**^**a**^	**− 7 to 0**	**21**	**42**	**63**	**84**	**105**	**126**	**195**	**285**	**375**	**……**
**Time windows (days)**	**--**	**±7**	**±7**	**±7**	**±7**	**±7**	**±7**	**±21**	**±21**	**±21**	**±21**
Informed consent	×										
Inclusion and exclusion criteria	×										
Survival state		×	×	×	×	×	×	×	×	×	×
General data	×										
History of diagnosis and treatment	×										
ECOG-PS	×										
Imaging examination	×		×		×		×	×	×	×	×
ORR			×		×		×	×	×	×	×
FACT-L scale	×	×	×	×	×	×	×	×	×	×	×
TCM symptoms scale	×	×	×	×	×	×	×	×	×	×	×
Tumor markers	×	×	×	×	×	×	×				
Blood, urine, stool routine examination	×	×	×	×	×	×	×				
Liver and renal function examination	×	×	×	×	×	×	×				
Electrocardiogram	×	×	×	×	×	×	×				
AEs		×	×	×	×	×	×	×	×	×	×
Compliance assessment		×	×	×	×	×	×	×	×	×	×
Drug combination		×	×	×	×	×	×	×	×	×	×

*Abbreviations: ECOG-PS* Eastern Cooperative Oncology Group performance status, *ORR* objective response rate, *FACT-L* Functional Assessment of Cancer Therapy-lung carcinoma, *TCM* traditional Chinese medicine, *AEs* adverse events

^**a**^At the end of each cycle in the treatment period and every 3 months in the follow-up period

#### Primary outcome

The primary outcome is overall survival (OS), from randomization to death.

#### Secondary outcomes

The secondary outcomes are as follow:

PFS: From randomization to occurrence of imaging-based disease progress (PD), which is assessed by the investigators.

ORR: Imaging examinations for the target lesions will be done at days 0, 42, 84, and 126 during the treatment period and every 90 days during the follow-up period. The tumor response is evaluated according to standards of Response Evaluation Criteria in Solid Tumors (RECIST) Version 1.1, rating as complete response (CR), partial response (PR), stable disease (SD), or PD. ORR is the ratio of participants rated as CR and PR to total cases.

QoL: Functional Assessment of Cancer Therapy-lung carcinoma (FACT-L) scale and TCM syndromes scale are used for the evaluation of QoL. The participants will answer the questionnaires in FACT-L independently with the assistance of investigators, covering physical, social and family, emotional, and functional statuses, as well as lung cancer module. The total score of FACT-L is calculated by adding up the scores of each item. TCM syndrome scale is designed to record the extent of several typical symptoms such as cough, phlegm, fatigue, shortness of breath, and loss of appetite. 0, 1, 2, and 3 points represent “none”, “mild”, “moderate”, and “severe”, respectively. QoL data are collected at baseline and in each visit.

Tumor markers: the commonest tumor markers, NSE and proGRP, are tested at days 0, 21, 42, 63, 84, 105, and 126 during the treatment period.

#### Safety assessment

The participants’ blood, urine and stool routine, liver and kidney function, and electrocardiogram were monitored at days 0, 21, 42, 63, 84, 105, and 126 during the treatment period. The AEs are categorized according to the National Cancer Institute's Common Terminology Criteria for Adverse Events (NCI-CTCAE) Version 4.03 and recorded overall the study.

### Register, ethical issues, and oversight

Before recruitment, we have registered the protocol in the Chinese Clinical Trial Registry (No. ChiCTR1900022991, 6 May 2019).

The study was ethically approved (2019L02-009-01) by the Research Ethical Committees at Beijing Hospital of the Traditional Chinese Medicine, Capital Medical University, and the other five participating sites. Now it is being conducted according to the principles of risks and benefits, privacy and confidentiality, informed consent, placebo using, etc., in the Declaration of Helsinki.

The oversight is in charge of Qihuang Contract Research Organization (CRO), through quarterly independently monitoring by clinical research associates (CRA) in terms of safety, quality, and progress of the study.

### Data management

PUCRI is responsible for data management. The data managers (DM) in PUCRI have built an EDC on the basis of CRF in REDCap system (Version 8.4.3) according to “data management and verification plan” drafted before. CRCs can type-in data to the EDC through their personal accounts. The errors or questions discovered by the system will be sent to the CRCs for answers. After all the data inputted without remaining questions, PI, sub-Is, and CRCs will lock the database within 4 weeks. Finally, the database will be exported by PUCRI and hand over to the statisticians. Only DMs have access to the final trial dataset.

### Statistical analysis

All data will be processed by a two-sided statistical test in SAS software 9.2 (the installation site’s authorization number: 11202165). *P*≤0.05 is defined as a statistical significance. Full analysis set (FAS), per-protocol set (PPS), and safety set (SS) will be created after data collection.

The statistical design is as follows:

For characteristics at baseline, continuous variables will be described with the use of mean (standard deviation) or median (quartile) based on the distribution. A T-test will be adopted if the normality and homogeneity of variance are satisfied; otherwise, the nonparametric test will be carried out. Categorical variables will be analyzed by the chi-square test or nonparametric test.

For OS and PFS, the Kaplan-Meier curve and the Log-rank test will be used to analyze the distribution of the survival function. And the factors selected in the univariate analysis will be brought into the Cox regression for further multivariate analysis.

For tumor response, the logistic regression or Ridit analysis will be used to compare the differences in ORR, while the proportion of CR, PR, SD, and PD will be compared by the CHM test because of the central effect.

For QoL and tumor markers, the T-test or RMANOVA will be used to compare the differences intra- or inter-groups if the two groups’ scores at baseline are similar. Or the covariance analysis will be used to correct for confounders if not.

For safety assessment, the AEs and their incidences should be recorded in detail.

## Discussion

To our knowledge, this is the first multicenter RCT aiming at long-term efficacy and prognosis of TCM granules with chemotherapy in ES-SCLC patients. Two published cohort research [15, 17] have reached an opposite conclusion about whether TCM decoctions can improve the PFS of ES-SCLC. Apart from the fact that both of their sample sizes were too small to be credible, they all made TCM as the only intervention but ignored the possibilities for patients choosing standard regimens simultaneously, which was not quite consistent with the present treatment status of ES-SCLC in China. Another RCT [18] of 80 SCLC cases demonstrated that TCM decoctions with standard EP could achieve a higher DCR versus EP alone. However, the study did not explain its randomization and blind. We suspect it may be an open-label trial, for no placebo was found. More crucially, the study did not stratify the limited and extensive stage in statistical analysis, which reduced the reference value of the results. Despite the lack of stringent design, the above studies have all affirmed the effectiveness of TCM on SCLC, especially in improving QoL. By contrast, our study does have several strengthens and innovations. First of all, this is a multicenter, randomized, single-blind, placebo-controlled clinical trial. The processes of grouping and single-blind are clear and well-documented. And the participants are from six grade-A tertiary hospitals in China, ensuring the representation of samples. The data are collected and managed on EDC, under the oversight of an independent monitoring organization, which is related to the credibility of our future findings. Secondly, our sample size is larger than previous similar studies. Thirdly, we focus on the role of TCM in prognosis, not just in reducing the toxicity or improving the QoL. The survival benefit is the key to judge TCM’s necessity in the treatment of ES-SCLC. And our study will solve the mystery. In addition, the multiple prescriptions in TCM regimens were designed for the common syndrome types of ES-SCLC, so as to realize the potentials and advantages of TCM syndrome differentiation.

The study still has some limitations as follows. First, the study was not designed as a double-blind trial. The traditional double-blind double-mimic method is not affordable and suitable for this study of multiple TCM prescriptions. Therefore, we finally chose single-blind which conforms to the complexity and feasibility of TCM and implemented it strictly to control the risk of reveals. Second, the assessment of OS may be influenced by confounders. Since the study intents to evaluate the long-term efficacy of TCM granules with chemotherapy, the TCM intervention period is designed as 4–6 cycles, synchronizing with chemotherapy. While in recent years, the second-line or later regimens are continuously refreshed by ICIs [19], anlotinib [20], lurbinectedin [21], and rovalpituzumab tesirine (Rova-T) [22]. As the study gives priority to the patient’s interests, the investigators should respect participants’ choices of positive treatment after PD. Thus, the second- and third-line regimen may become vital factors for prognosis. As a remedy, we require the investigators to keep detailed records of regimen participants using after PD and provide them to statisticians to build a more realistic and reasonable regression model.

In conclusion, the study’s result will provide a high-level evidence for TCM granules using with chemotherapy on the first-line treatment of ES-SCLC.

## Trial status

The trial is conducted according to the protocol (Version 2.0, 4 June 2019). It is currently enrolling participants. The recruitment began on 20 June 2019 and was expected to be completed on 19 June 2021. The first participant was enrolled on 4 September 2019.

## Supplementary Information

**Additional file 1.** Supplement 1. Compositions and dosages of TCM granules.

## Data Availability

Not applicable.
