# Supplementary material for: Effects of traditional Chinese medicine combined with chemotherapy for extensive-stage small-cell lung cancer patients on improving oncologic survival: study protocol of a multicenter, randomized, single-blind, placebo-controlled trial
Source: Trials. 2021 Jul 8;22:437. doi: 10.1186/s13063-021-05407-1 (PMC8265049; doi:10.1186/s13063-021-05407-1)
Supplement: Supplementary file 1 — Additional file 1. Supplement 1. Compositions and dosages of TCM granules. [file 13063_2021_5407_MOESM1_ESM.docx]

**Supplement 1.** Compositions and dosages of TCM granules

| **Recipes/herbs** | **Latin names** | **Dosages (g)** |
| --- | --- | --- |
| **TCM Recipe 1** |  |  |
| Huang Qi | Astragall Radix | 40 |
| Bei Sha Shen | Glehniae Radix | 20 |
| Mai Dong | Ophiopogonis Radix | 15 |
| Bai He | Lilii Bulbus | 15 |
| Xuan Shen | Scrophulariae Radix | 15 |
| Zhe Bei Mu | Fritillariae Thunbergii Bulbus | 15 |
| Ku Xing Ren | Armeniacae Semen Amarum | 10 |
| Ban Zhi Lian | Scutellariae Barbatae Herba | 20 |
| Bai Hua She She Cao | Hedyotis Diffusa | 20 |
| **TCM Recipe 2** |  |  |
| Huang Qi | Astragall Radix | 40 |
| Dang Shen | Codonopsis Radix | 15 |
| Bai Zhu | Atractylodis Macrocephalae Rhizoma | 15 |
| Fu Ling | Poria | 15 |
| Qing Ban Xia | Pinelliae Rhizoma Praeparatum cum Alumine | 10 |
| Chen Pi | Citri Reticulatae Pericarpium | 15 |
| Jie Geng | Platycodonis Radix | 15 |
| Yi Yi Ren | Coicis Semen | 30 |
| Zhe Bei Mu | Fritillariae Thunbergii Bulbus | 15 |
| Ku Xing Ren | Armeniacae Semen Amarum | 10 |
| **TCM Recipe 3** |  |  |
| Mai Dong | Ophiopogonis Radix | 15 |
| Di Huang | Rehmanniae Radix | 20 |
| Mu Dan Pi | Moutan Cortex | 15 |
| Shan Zhu Yu | Corni Fructus | 10 |
| Wu Wei Zi | Schisandrae Chinensis Fructus | 10 |
| Zhi Mu | Anemarrhenae Rhizoma | 15 |
| Zhe Bei Mu | Fritillariae Thunbergii Bulbus | 15 |
| Gua Lou | Trichosanthis Fructus | 20 |
| Xia Ku Cao | Prunellae Spica | 15 |
| **TCM Recipe 4** |  |  |
| Dang Gui | Angelicae Sinensis Radix | 15 |
| Chi Shao | Paeoniaeradix Rubra | 15 |
| Xian He Cao | Agrimoniae Herba | 15 |
| Yi Yi Ren | Coicis Semen | 15 |
| Xia Ku Cao | Prunellae Spica | 15 |
| Yan Hu Suo | Corydalis Rhizoma | 20 |
| Zhi Qiao | Aurantii Fructus | 12 |
| Zhe Bei Mu | Fritillariae Thunbergii Bulbus | 15 |
| E Zhu | Curcumae Rhizoma | 10 |
| **TCM Recipe 5** |  |  |
| Chen Pi | Citri Reticulatae Pericarpium | 15 |
| Qing Ban Xia | Pinelliae Rhizoma Praeparatum cum Alumine | 10 |
| Fu Ling | Poria | 15 |
| Bai Zhu | Atractylodis Macrocephalae Rhizoma | 15 |
| Dang Shen | Codonopsis Radix | 15 |
| Yi Yi Ren | Coicis Semen | 30 |
| Ku Xing Ren | Armeniacae Semen Amarum | 10 |
| Gua Lou | Trichosanthis Fructus | 20 |
| Huang Qin | Scutellariae Radix | 15 |
| Lu Gen | Phragmitis Rhizoma | 20 |
| Lian Qiao | Forsythiae Fructus | 15 |
| Yu Xing Cao | Houttuyniae Herba | 20 |
| Ban Zhi Lian | Scutellariae Barbatae Herba | 20 |
| Bai Hua She She Cao | Hedyotis Diffusa | 20 |
| **TCM Recipe 6** |  |  |
| Feng Fang | Vespaenidus | 5 |
| Quan Xie | Scorpio | 5 |
| Tu Bie Chong | Eupolyphaga Steleophaga | 6 |
| Xia Ku Cao | Prunellae Spica | 15 |
| Mao Zhao Cao | Ranunculi Ternati Radix | 20 |
| Gui Jian Yu | Euonymus Alatus | 15 |
| Ban Zhi Lian | Scutellariae Barbatae Herba | 30 |
| Bai Hua She She Cao | Hedyotis Diffusa | 30 |
| Dang Shen | Codonopsis Radix | 15 |
| Bai Zhu | Atractylodis Macrocephalae Rhizoma | 15 |
| Fu Ling | Poria | 15 |
| Huang Qi | Astragall Radix | 50 |
